# Substitution—A Department Store Found Guilty

**Published:** 1904-06

**Authors:** 


### Substitution—A Department Store Found Guilty.

WE HAVE all noticed the evolution of the department store from the country general store; from the little shop where one can get pretty nearly anything coming within the needs of man from a corn plaster to a set of false teeth, to the magnificent palaces of industry and trade of the big city of today, in which everything is found,—dry goods, and shiny silks and shimmering satins; rare jewels and pellucent pearls; green groceries, canned colics, shoes and slippers and jeans and jerseys; automobiles and art works; paints and powders and drugs, and where there is even a hospital ward room perfectly equipped and attended by a coolly-clad nurse in cap and uniform, with a "near-by physician in attendance." We, in our ignorance of purely commercial business may have marveled at the perfection of the organisation, and at the same time been aware of the self-evident fact that in many of these carry-alls of commerce the sale of proprietary medicines was carried on "below cost," and of the other painfully apparent fact that substitution reared its ugly head high amid all the glories and beautiful bewitcheries of the fine things of feminine fancy.

In New York City this evil has been more arrogant than in Buffalo, and while the owners of the proprietary preparations have been endeavoring to put a stop to substitution, apparently the most vigorously aggressive action was taken when the Siegel-Cooper Company, of New York, began advertising a preparation known as "pepto-manganate." This was so evident a substitution and such a palpably bold and brazen attempt at larceny that the M. J. Breitenbach Company, owners of Gude's pepto-mangan, brought action against the dry goods concern for a permanent injunction, restraining it from using the words "pepto-manganate" or any other words simulating "pepto-mangan," and for an accounting of the sales of the substituted product; and for an order requiring the destruction of all material, bottles, labels and other printing used in the manufacture or sale of the substituted product. The action was tried before Justice Bischoff of the Supreme Court and resulted in a sweeping order, granting all the relief asked and all the claims set up in the complaint of the Breitenbach company. This decision is far reaching. It will have the effect of curbing the commercial activity of the department store insofar as the sale of substitutes for well-known and beneficial preparations is concerned. Justice Bischoff makes very strong his position as regards the invasion and violation of trade-mark and trade-name rights.

It was a bold stroke of business enterprise for the Siegel-Cooper Company to enter the proprietary field, but they committed the fatal error of claiming a relationship which did not exist. During the course of the action there was no question raised regarding the purity or the efficacy of the Siegel-Cooper Company preparation; that point was not on trial. Legally viewed the case merely involved the invasion of a trade-mark or trade-name right; morally it was the evil of substitution which was on trial—and decency and honesty were victorious.

The M. J. Breitenbach Company is deserving of praise and congratulation for the able and vigorous manner in which the fight was carried on and won.

